# Scope of a weekly infection control team rounding in an acute-care teaching hospital: a pilot study

**DOI:** 10.1186/s13756-020-00787-6

**Published:** 2020-08-15

**Authors:** Yeon Su Jeong, Jin Hwa Kim, Seungju Lee, So Young Lee, Sun Mi Oh, Eunjung Lee, Tae Hyong Kim, Se Yoon Park

**Affiliations:** 1 Infection Control Team, Soonchunhyang University Seoul Hospital, Seoul, Republic of Korea; 2 Division of Infectious Diseases, Department of Internal Medicine, Soonchunhyang University College of Medicine, Seoul, Republic of Korea; 3 Chungbuk National University College of Medicine, Cheongju, Republic of Korea

**Keywords:** Infection control and prevention, Cross infection, Catheter-related infections, Surgical wound infection, Infection safety practices

## Abstract

Regular and well-organized inspection of infection control is an essential element of an infection control program. The aim of this study was to identify the functional scope of weekly infection control team rounding (ICTR) in an acute care hospital. We conducted weekly ICTR between January 18 and December 26, 2018 to improve the compliance to infection control and prevention measures at a 734-bed academic hospital in the Republic of Korea and analyzed the results retrospectively. We categorized the results into five groups: “well maintained,” “improvement needed,” “long-term support, such as space or manpower, needed,” “not applicable,” or “could not be observed”. A total of nine categories and 85 sub-elements of infection control and prevention practices were evaluated. The median number of infection control team (ICT) visits per department was 7 (interquartile range [IQR]: 6–7). The ICT assessed a median of 16 elements (IQR: 12–22), and a total of 7452 results were obtained. Of those, 75% were monitored properly, 22% were “not applicable”, and 4% were difficult to observe. The most common practices that were difficult to observe were strategies to prevent catheter-related surgical site infections, pneumonia, and occupationally acquired infections as well as injection safety practices. Although the ICTR was able to maintain regular visits to each department, further strategies beyond regular ICTR are needed to reduce category of “could not observed”. This pilot study may provide an important reference for institutional infection prevention practices as it is the first study to investigate the functional coverage of ICTR.

## Background

Healthcare-associated infections (HAIs) significantly contribute to patient mortality and morbidity [1]. Leadership rounding for HAI prevention has emerged as a method to maintain and develop HAI-preventive practices in healthcare units [2, 3]. Previous studies have found that leadership rounding for strategic discussions, including prevention of catheter-associated urinary tract infections [4], surgical site infection (SSI) [5], and central line-associated bloodstream infections (CLABSI) [2, 4], has a dramatically positive effect on reducing HAI rates. Infection control activities are diverse and complex as they include practices that protect healthcare workers, visitors, trainees, and patients from infections. Since 2017, the government of the Republic of Korea has been providing financial support to medical institutions that enforce weekly assessments of adherence to infection prevention protocols, known as infection control team rounding (ICTR). This method can be useful in monitoring infection control practices in different hospital departments.

Several studies related to performing leadership rounding to reduce HAIs have been conducted [2–5]. However, there is a limited amount of information regarding the scope of regular ICTR that comprehensively examines all categories of infection control activities in different hospital departments. Therefore, this study aims to investigate the functional scope of ICTR on infection control activities by examining the applicability of each item on the rounding checklist in a real hospital setting.

## Methods and materials

This study was conducted at Soonchunhyang University Seoul Hospital, a 734-bed academic hospital in Republic of Korea. In this study, the results of the ICTR performed in our hospital between January and December 2018 were analyzed retrospectively. Since January 2018, the rounding results were categorized into the following five groups: “well maintained,” “improvement needed,” “long-term support, such as space or manpower, needed,” “not applicable,” or “could not be observed.” The classification criteria were determined on the basis of a consensus reached by infection control team members.

The purpose of conducting the rounding was to improve compliance with infection control measures and determine the categories that needed improvement, or financial or administrative support. The monitoring team included five infection practitioners and four infectious diseases physicians. Each infection control rounding required approximately 2 hours to complete. The study was approved by the Institutional Review of Board of our hospital.

The following nine categories of infection control and prevention items were included: (1) hand hygiene (8 items); (2) safety injection practice (9 items); (3) isolation (10 items); (4) strategies to prevent occupationally acquired infections (6 items); (5) practices to prevent catheter-related (central, urine catheter), surgical site infection and pneumonia (16 items); (6) decontamination, disinfection, and sterilization (19 items); (7) linen and laundry management (6 items); (8) environmental prevention of infection (8 items); and (9) maintaining negative/positive pressure (3 items) as shown in Supplemental Table 1 [6].

During each ICTR, at least two categories were monitored. The different categories including practices to prevent catheter-related (central, urine catheter), surgical site infection, and pneumonia (5), and decontamination, disinfection, and sterilization (6) were monitored separately as they required a detailed review of a large number of practices. The schedule and inspection items were communicated to each department in advance. The inspection was conducted through direct observation or an interview, and the results were entered by the infection control team members.

## Results

**Fig. 1:**
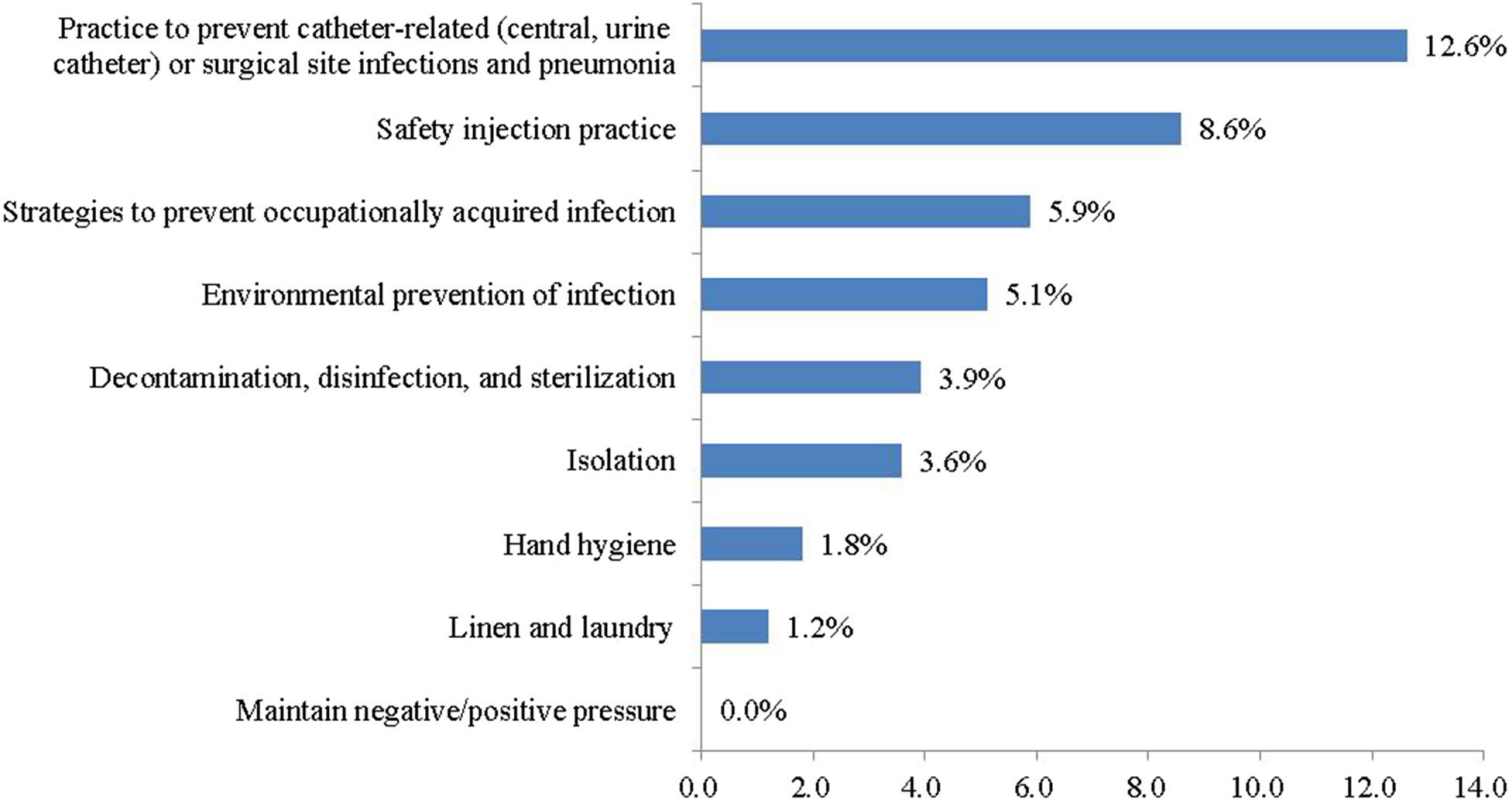
Categories of practices that were difficult to observe through regular infection control team rounding

A total of nine categories with 85 infection control and prevention items were observed. During the study period, ICTR was performed a total of 45 times in 36 departments. Furthermore, a median of 7 (interquartile range [IQR]: 6–7) ICTR visits were performed in each department and a median of 16 practices (IQR: 12–22) were assessed during the ICTR, and 7452 results were recorded. Of those, 74.6% (5558) were observed: “well-maintained” practices constituted 69.9% (5208), “improvement needed” accounted for 4.4% (331), and “long-term support needed” accounted for 0.3% of all practices (19). A total of 1601 (21.5%) results were “not applicable” and 293 (3.9%) were difficult to observe through ICTR (Table 1). Among applicable practice results, the most common practices that were difficult to observe were strategies to prevent catheter-related, surgical site infections, and pneumonia (12.6%, 68/538) as well as injection safety practices (8.6%, 65/758) and strategies to prevent occupationally acquired infections (6.4%, 37/578) (Fig. 1).

**Table 1:** Results of infection control team rounding

Categories of practices	Well maintained (%)	Improvement needed (%)	Long-term support needed (%)	Not applicable (%)	Could not be observed (%)	Total
Hand hygiene	936 (93.6)	46 (4.6)	0	0	18 (1.8)	1000
Safety injection practice	664 (75.0)	28 (3.2)	1 (0.1)	127 (14.4)	65 (7.3)	885
Isolation	391 (57.5)	12 (1.8)	0 (0)	262 (38.5)	15 (2.2)	680
Strategies to prevent occupationally acquired infection	506 (80.6)	35 (5.6)	0	0	37 (5.9)	628
Practice to prevent catheter-related (central, urine catheter) or surgical site infections and pneumonia	451 (48.6)	19 (2.0)	0 (0)	390 (42.0)	68 (7.3)	928
Decontamination, disinfection, and sterilization	1349 (69.6)	128 (6.6)	12 (0.6)	388 (20.0)	61 (3.1)	1938
Linen and laundry	451 (78.7)	33 (5.8)	6 (1.0)	77 (13.4)	6 (1.0)	573
Environmental prevention of infection	403 (68.1)	24 (4.1)	0	142 (24.0)	23 (3.9)	592
Maintain negative/positive pressure	57 (25.0)	6 (2.6)	0	165 (72.4)	0	228
Total	5208 (69.9)	331 (4.4)	19 (0.3)	1601 (21.5)	293 (3.9)	7452

## Discussion

Our study showed that the majority of HAI prevention practices implemented at our hospital can be monitored through regular ICTR. As a result of the ICTR and the assessment that followed, we found that the reasons behind underperformance in some infection control activities lie at the individual and organizational levels. Moreover, we identified a limitation of regular ICTR: some infection control activities are not applicable to patients and cannot be monitored through regular ICTR. Infection prevention practices associated with preventing breathing device-related infection and SSIs were found to be more difficult to monitor. Considering these findings, it is necessary to revise the protocol to ensure that all infection control activities can be practiced and monitored correctly in accordance with the manual.

Although several previous studies related to ICTR have been conducted, these studies investigated the benefits of incorporating leadership rounding into infection prevention and focused on the association between infection control activities with rounding and one specific type of infection [2, 4–8]. These assessments indicated that ICTR was successful in the studied context; nonetheless, these studies lack a comprehensive perspective on the mechanisms of rounding. In contrast, our study evaluated monitoring of infection control activities based on the entire infection prevention protocol, with a grade-based assessment of infection control activities. Thus, we assessed infection control activities related to a variety of infections, rather than focusing on one specific infection. As a result, we could delineate the functional coverage of ICTR monitoring and applicability of each infection prevention activity. Notably, this method of assessment highlights the practices that require organizational decisions and changes beyond individual performance to result in improvement, i.e., we concluded that implementing improvements in linen and laundry management requires an additional budget.

Our pilot study is the first study to specify items that require monitoring for hospital-associated infection control. To our knowledge, there is no previous study that suggests a comprehensive list of items that require monitoring, which is necessary for a structural improvement of the ICTR system. Although we could not validate the grounds for selecting each item because of the absence of previous studies, the list consists of items necessary for infection control, which the South Korean government also applies to assess medical institutions [9]. Therefore, medical institutions can refer to our study in order to ensure compliance with the infection prevention accreditation criteria. Furthermore, our study distinguishes between items that can be monitored via organizational ICTR of a particular department and those that cannot. In case of the latter group, additional time and human resources or an alternative approach to monitoring may be required. For example, institutions may decide to designate one or two people to be responsible for the entire monitoring process in a particular department, rather than sharing responsibility across departments. A suitable observation method should be determined given the specific circumstances of each medical institution.

Despite several merits, our study has some limitations. First, this study was conducted in a single South Korean hospital. As every healthcare center has its own unique characteristics, the results obtained in this study may not be extrapolated to other hospitals. Second, notifying each department in advance about the schedule and inspection items may have resulted in a Hawthorne effect. Nonetheless, we expect to see improvements in infection control compliance rates providing we continue monitoring in the same fashion.

In this study, we investigated the challenges related to ICTR based on observed performance levels. Hospitals should focus on potentially inappropriate practices and revise the protocol to expand the functional scope of the ICTR once a problem has been identified. Furthermore, each medical institution should determine the interventions that can be implemented at an individual level and those that require additional organizational support.

## Supplementary information

**Additional file 1: Supplemental Table 1.** Checklists for infection control team rounding.

## Data Availability

All data generated or analyzed during this study are included in this published article and its supplementary files.

## Abbreviations

HAIs: Healthcare-associated infections
SSI: Surgical site infection
ICTR: Infection control team rounding
IQR: Interquartile range
