# Supplementary material for: Scope of a weekly infection control team rounding in an acute-care teaching hospital: a pilot study
Source: Antimicrob Resist Infect Control. 2020 Aug 15;9:123. doi: 10.1186/s13756-020-00787-6 (PMC7428421; doi:10.1186/s13756-020-00787-6)
Supplement: Supplementary file 1 — Additional file 1: Supplemental Table 1. Checklists for infection control team rounding. [file 13756_2020_787_MOESM1_ESM.docx]

**Supplemental Table 1.** Checklists for infection control team rounding

| Domain | Checklist Items | Method of Monitoring |
| --- | --- | --- |
| Hand hygiene | An alcohol-containing gel is placed in front of the ward, bed, nurse room, and treatment room, and delivers smoothly upon light touch | Observation |
|  | Expiration date of the alcohol-containing gel is correctly written on the container and the gel is replaced before its expiration date | Observation |
|  | Appropriate hand hygiene practice method is posted near the washstand | Observation |
|  | Liquid soap is filled and comes out smoothly | Observation |
|  | A poster describing hand hygiene timing, methods, and outcomes is posted and readable (not damaged) | Observation |
|  | All hospital staff are aware of the department's hand hygiene compliance rate and target rate | Interview |
|  | Staff are notified of means for sharing the hand hygiene compliance rate (e.g., poster, groupware board, B1 board, paper) | Interview |
|  | All hospital staff are aware of the department's campaign for increasing the hand hygiene compliance rate | Interview |
| Safety injection practice | The injection preparation zone is kept clean | Observation |
|  | Sanitizing tissues are prepared in the injection preparation zone | Observation |
|  | Syringes, needles, and solution bags are stored in package-form (unpacked immediately before use) | Observation |
|  | Multiple-use injection solutions are stored clean in a place far from the injection zone and the first use date is recorded on the container | Observation |
|  | No disposable syringe leftovers are present (e.g. infusion solution, lidocaine) | Observation |
|  | The drug is prepared immediately before the injection, and is injected, at latest, within an hour | Observation |
|  | Injection (solution) is not filled into the syringe in advance | Observation |
|  | Syringes that contain drugs are not carried in pockets | Observation |
|  | Injection rubber stopper, cleaved ampoule, syringe, and/or catheter hub are sanitized after use | Observation |
| Isolation | Be aware of how to recognize patients with infectious diseases (e.g., with digital drop, sticker, and label) | Interview |
|  | Know isolation procedures, including their application and cancellation (ISO, ISODC, Isolation notice) | Interview |
|  | Medical supplies, including linens used in the isolation room, are carried in a plastic bag | Observation/Interview |
|  | Posters are posted outside and inside the isolation room, and personal protective gear is prepared | Observation |
|  | Personalized medical device, hand sanitizer, and isolation medical waste container are prepared in the isolation room | Observation |
|  | Be aware of procedures for accessing the classified isolation room (personal protective gear wearing and hand hygiene practice) | Observation/Interview |
|  | Do not reuse personal protective equipment | Observation |
|  | Notify the destination (department) of isolated patient transfer via offline phone | Interview |
|  | Change the patient gown when an isolated patient with multidrug-resistant bacteria is transferred (or wear a disposable gown, and replace the bed sheet when moving the patient to another bed) | Observation |
|  | Practice management of the isolated room in accordance with instructions (everyday, practice is followed by completing the checklist) | Observation/Interview |
| Strategies to prevent occupationally-acquired infection | A line should appear at the ¾-filled point of acute medical waste containers; do not dispose of waste when the contained is filled above this line | Observation |
|  | Use safety needles for patients with blood-mediated pathogens or sources of infections | Observation/Interview |
|  | Be aware of protocols for reporting and coping with accidental exposure to infection (First aid → Digital report → Offline contact) | Interview |
|  | Attach a ‘B’ sticker if blood-mediated pathogens or sources of infection are found during blood cultivation | Observation/Interview |
|  | Dispose of sharp devices immediately after use | Observation |
|  | Be aware of means to share the current accidental exposure to infection (e.g. Poster, groupware board, B1 board, paper) | Interview |
|  | Be aware of the contents of the checklist for disposition to high-risk departments | Interview |
|  | Manage the immune status recordings (lists), including the vaccination history of the department staff | Observation/Interview |
| Practices to prevent catheter-related (central, urine catheter) or surgical site infection and pneumonia | Be aware of protocols for central vein cannulation and complete the checklist (e.g. wearing a cap, mask, sterilized gown, sterilized linen, sterilized globe, 2% CHG) | Observation/Interview |
|  | Observe and record the state of the central vein cannulation region | Observation |
|  | Be aware of and record the sanitizing cycle of the central vein cannulation | Observation |
|  | Record the injection date near the peripheral vein cannulation region | Observation/Interview |
|  | Use a sterilized catheter and disposable saline solution per each suction | Observation |
|  | Keep the tracheostomy region dry | Observation |
|  | Sterilize and replace the tracheostomy inner tube | Observation/Interview |
|  | Follow the protocol for cleansing the nebulizer (high-level sanitizing) | Observation/Interview |
|  | The expiration date (from the day of being connected) of the merchandized disposable oxygen container is recorded | Observation |
|  | Act in accordance with the management protocol in case of using a reusable oxygen container (exchange saline solution on a daily basis, high-level sanitizing, sanitation cycle, compliance with expiration date, clean storage) | Observation/Interview |
|  | After the ambu-bag is used, it is washed and sterilized properly, and then stored clean | Observation |
|  | Use a 0.5% CHG sanitizer before injection of the urinary tract catheter | Observation/Interview |
|  | The urine bag management protocol sticker is attached | Interview |
|  | Comply with the urine bag management protocol (e.g. place under the bladder, above the floor, and maintain occlusion) | Observation |
|  | Use the urine container individually and keep it clean | Observation |
|  | Be aware of the methods for collection of urine sample from patients with urinary tract catheter | Observation/Interview |
|  | In need of depilation, use a clipper (use of a razor is forbidden) | Observation/Interview |
| Decontamination, disinfection, and sterilization | Do not reuse disposable items | Observation |
|  | Use an unpacked dressing package within an hour, and keep it away from dirt and/or water | Observation |
|  | Wear personal protective gear (shield mask, globe, medical gown, waterproof shoes) while washing | Observation/Interview |
|  | Wash the items, which are sterilized with disinfectants, with sterilized saline solution | Observation/Interview |
|  | Make a distinction between clean and contaminant areas in a cart (e.g put samples and drugs in separate places) | Observation |
|  | Store sterilized items and clean items in separate places, and keep them from exposure to dirt | Observation |
|  | First in, First out of sterilized items | Observation |
|  | There are no exposed or expired sterilized/anti-septic items | Observation |
|  | The opening and expiration dates of the sanitizer are recorded | Observation |
|  | Be aware of and label the usage and expiration dates of the diluted sanitizer/detergent | Observation |
|  | The expiration date of the disinfection can (1 day) is maintained | Observation |
|  | Label the sterilizing date and the date of first use on the medical device being sterilized | Observation |
|  | Sterilize the sedimentation container of the medical device when the diluting solution is replaced | Observation/Interview |
|  | Comply with the replacement cycle of the forceps jar | Observation/Interview |
|  | Seal the medical supply used by CJD patients in a plastic bag, without washing, and then return it after labeling "Danger: CJD contamination" | Observation/Interview |
|  | Practice sterilization of the medical device in accordance with the appropriate protocol for each type of medical device | Observation/Interview |
|  | Clean the tray with sanitizer while aiding the patient | Observation/Interview |
|  | Use a disposable blood-sugar test pen (if it is in common use, sterilize it before each use) | Observation/Interview |
|  | Replace the cap every time anyone uses a tympanic thermometer | Observation/Interview |
| Linen and laundry management | Set the linen storage and laundry room in the secluded area | Observation |
|  | Cover the linen while carrying it to different departments | Observation |
|  | There is no linen stored while the linen room is open | Observation |
|  | Keep used laundry from overflowing from the collection bag | Observation |
|  | Label contaminated laundry on the collection bag | Observation |
|  | Sanitize the contaminated laundry storage room everyday (complete the checklist) | Observation |
| Environmental prevention of infection | Replace curtains on a regular basis and record the replacement date (Replacement cycle: two times per year, or whenever contaminated) | Observation |
|  | Maintain the cleanliness of the blinds (remove dirt every two months, and replace seriously contaminated blinds) | Observation |
|  | Practice environmental control before leaving the room and complete the checklist | Observation |
|  | Keep the containers free from impairments and dirt | Observation/Interview |
|  | Sterilize the refrigerator on a regular basis (1 time per week) | Observation/Interview |
|  | Mops are distributed to each area | Observation |
|  | Put the mop (rag pointing towards the ground) in air | Observation |
|  | Maintain cleanliness of the janitor room and do not store items other than those used for cleaning | Observation |
| Maintaining negative/positive pressure | Maintain negative/positive pressure (equal to or above 2.5pa, ward > other rooms) | Observation |
|  | Negative/positive pressure checklist is completed in accordance with the rules | Observation |
|  | Regular maintenance inspection is practiced | Observation |
